# Assessing eating context and fruit and vegetable consumption in children: new methods using food diaries in the UK National Diet and Nutrition Survey Rolling Programme

**DOI:** 10.1186/1479-5868-9-126

**Published:** 2012-10-18

**Authors:** Tsz Ning Mak, Celia J Prynne, Darren Cole, Emily Fitt, Caireen Roberts, Beverley Bates, Alison M Stephen

**Affiliations:** 1 MRC Human Nutrition Research, Elsie Widdowson Laboratory, 120 Fulbourn Road, CB1 9NL, Cambridge, UK; 2 National Centre for Social Research, 35 Northampton Square, London, EC1V 0AX, UK

**Keywords:** Eating context, National Diet and Nutrition Survey, Fruit and vegetables, Eating occasion, Ecological Momentary Assessment, Children

## Abstract

**Background:**

Eating context is the immediate environment of each eating occasion (EO). There is limited knowledge on the effects of the eating context on food consumption in children, due to the difficulty in measuring the multiple eating contexts children experience throughout the day. This study applied ecological momentary assessment using food diaries to explore the relationships between eating context and fruit and vegetable consumption in UK children.

**Methods:**

Using 4 d unweighed food diaries, data were collected for 642 children aged 1.5-10y in two years of the UK National Diet and Nutrition Survey (2008–2010). Participants recorded all foods and drinks consumed at each EO, where and with whom the food was consumed, whether the TV was on and if eaten at a table. Mixed logistic regression and mixed multinomial logistic regression were used to calculate associations between eating contexts and fruit and vegetables (FV) consumed by quartiles.

**Results:**

Of 16,840 EOs, 73% took place at home and 31% with parents only. Frequency of eating alone and with friends increased with age. Compared to eating at home, children aged 1.5-3y were more likely to consume fruit at care outside home (>10-50g OR:2.39; >50-100g OR:2.12); children aged 4-6y were more likely to consume fruit (>50-100g OR:3.53; >100g OR:1.88) and vegetables at school (>30-60g OR:3.56). Compared to eating with parents only, children aged 1.5-3y were more likely to consume fruit with friends (>10-50g OR:2.69; >50-100g OR:3.49), and with carer and other children/others (>10-50g OR:2.25); children aged 4-6y were more likely to consume fruit (>50-100g OR:1.96) and vegetables with friends (>30-60g OR:3.56). Children of all ages were more likely to eat vegetables when the TV was off than on and at a table than not at table.

**Conclusions:**

The use of food diaries to capture multiple eating contexts and detailed fruit and vegetable consumption data was demonstrated at a population level. Higher odds of FV consumption were seen from structured settings such as school and care outside home than at home, as well as when eating at a table and the TV off. This study highlights eating contexts where provision of fruit and vegetables could be improved, especially at home. Future research should take eating context into consideration when planning interventions to target children’s food consumption and eating behaviour.

## Background

Fruit and vegetable (FV) consumption is an established positive dietary behaviour with beneficial effects on health; consumption is linked to reduced risk of diet related diseases such as cardiovascular disease [1,2] and diabetes [3]. Children are a particular target group for promotion of increased consumption, as evidence suggests diet and eating habits in childhood are likely to track and persist into adulthood [4], and that protective properties of fruits and vegetables may need to be present in early stages of life to be effective in adulthood in preventing these diseases [5]. Thus, children who consume higher quantities of FV during childhood are likely to be healthier in later life with lower risk of developing diet-related chronic conditions [6,7].

The food environment is an important determinant of dietary behaviour. Recent studies have focussed on the availability and accessibility of FV in the built environments such as neighbourhoods [8,9] and schools [10,11], as well as home food environment [12-15]. However, little is known about the relationship between the immediate meal environment and FV consumption in children, particularly across multiple meal locations and social contexts. Children experience a range of social meal settings throughout the day, such as school, formal and informal care, home, as well as eating with different people under these settings. The provision of food for children is likely to be different in different locations and consumption may differ by the other people present at eating occasions. Moreover, external influences such as television and behaviours like eating at the table during meals may influence consumption. Previous studies suggest that TV watching may encourage junk food consumption [16,17] whilst eating shared family meals may encourage healthy food consumption [18]. It is therefore important to assess various eating contexts in relation to consumption in children, and identify specific contexts that may encourage or discourage positive healthy eating behaviours. This can stimulate parents and carers to consider and potentially change their children’s eating environment such as to limit eating in front of the TV and to eat together at the table, as well as to enable researchers in targeting specific eating contexts in future health promotion interventions.

Measuring the eating context is difficult, particularly at a population level. Previous research has used questionnaires to measure a number of factors that describe the home environment, such as food availability, family meal and screen time [13,18-22]; such methods give indications of the usual frequency of certain exposures but not a detailed account of the context at the time of eating. A number of studies have investigated school meal surroundings and consumption using direct observation methods [10,23]. However, these on-site observations are only suitable for capturing single meals at specific community settings such as schools, and are likely to be burdensome and costly. A possible solution to overcome these limitations, and to capture both multiple eating contexts and to record detailed dietary data at the same time, is the use of Ecological Momentary Assessment (EMA). This approach is useful for collecting real-time data in natural environments over time and across contexts; however it has mainly been applied in physical activity research and clinical psychological studies [24,25]. To our knowledge, EMA has not been used to study the eating context at a population level in nutritional research. Our aim was to combine EMA with dietary assessment using food diaries, to study the association between eating contexts and fruit and vegetable consumption at the eating occasion level in a national representative sample of UK children. The eating context was defined by four elements: the location of the eating occasion, who was present, whether the television was on, and if consumed at a table. These four elements were selected as they were easy to record without extra burden on the participants.

## Methods

### Study sample and design

The data used were from 642 children aged 1.5 to 10 years from Year 1 (February 2008 to April 2009) and Year 2 (April 2009 to April 2010) of the National Diet and Nutrition Survey (NDNS) Rolling Programme. NDNS assesses food consumption, nutrient intakes and nutritional status of free living people aged 1.5 years and over in the UK. A detailed description of the sampling procedure and design of the rolling programme has been reported elsewhere [26]. The sampling is based on a random selection of postcode sectors throughout the UK, and a number of addresses per sector are visited by interviewers to invite participation, following an initial information letter. Up to one adult and one child could be selected from each participating household and a child “boost” of addresses where only children were recruited is included to ensure an even number of adults and children, since many households have no children [26]. NDNS was conducted according to the guidelines laid down in the Declaration of Helsinki and all procedures involving human subjects were approved by the Oxfordshire A Research Ethics Committee. Written informed consent was obtained from all participants [26].

### Dietary assessment

At the initial interviewer visit, parents were given instructions to record all food and beverages consumed by the participating child in and out of the home over four consecutive days using unweighed food diaries. Recording for three days or more was considered fully productive. Parents were asked to seek help from short term carers, adult friends and relatives, and teachers with the recording process if children had been away from parents [27]. Portion sizes were estimated using household measures and using weights from food package labels obtained online or by purchase, or provided by participants. For homemade dishes, recipes were recorded separately with detailed description of ingredients, quantities and cooking methods. Further description and justification of the dietary assessment methods used in the NDNS rolling programme have been reported previously [26,27]. At the final interviewer visit, the food diaries were checked for missing information and added further detail if possible before they were returned to Medical Research Council- Human Nutrition Research for coding. The diaries were coded by trained coders using the in-house dietary assessment software, Diet In Nutrients Out (DINO), with nutrient values provided by the UK NDNS Nutrient Databank (NDB).

### Disaggregation of fruit and vegetable consumption

To quantify the consumption of FV more accurately, disaggregation was undertaken to account for consumption from composite dishes containing FV, as well as discrete portions. This is particularly important in studying FV consumption in younger children, as there is a wide variety of food products intended for children e.g. toddler ready meals, snacks and dairy products such as yoghurts, that contain a mixture of fruit and/or vegetables as well as other ingredients. Portion sizes of discrete FV items were determined using FSA’s reference book on Food Portion Sizes [28]. The method adopted to disaggregate food codes in NDNS has been described fully in a previous paper [29].

### Assessment of the eating context at meal

In the food diaries, each recording day was divided into seven timeslots (6-9am, 9-12pm, 12-2pm, 2-5pm, 5-8pm, 8-10pm and 10pm-6am) to reflect the time for various meals and snacks. For every episode of eating, parents were asked to record in the corresponding timeslot where their child was, who the child was with, whether TV was on and if the child was eating at a table, as well as the actual time of consumption (see diary extract in Additional file 1: Appendix 1). In some timeslots, children had multiple eating occasions. For each food entered into DINO, codes were allocated to describe “Where” the food was consumed, “With whom”, “TV on” and “At a table”. For children aged 1.5-10 years, responses for “Where” were initially coded into 29 subcategories and “With whom” coded into 12 subcategories. These subcategories were further collapsed into fewer categories to give better distribution of data (Additional file 2: Appendix 2). For children aged 1.5-3y, “Where” was condensed into “At home”, “Friend’s or relative’s house”, “Care outside home”, “Other eateries”, and “Other places” categories. For school children aged 4-10y, a category “At school” was added to account for eating occasions that took place within school. For children aged 7-10y, the category “Care outside home” recoded with “Other places” due to very few eating occasions taking place at care outside home in this age group. The categories for the “With whom” variable were condensed to “Parents only”, “Parents and siblings”, “Adult relatives and friends”, “Carer and other children/others”, “Alone” and “Friends”. Both “Care outside home” and “Carer and other children/others” refer to formal care settings in this study. “TV on” and “Sitting at a table” were coded as “Yes” or “No”. Where participants did not record answers in any of these four eating context variables, they were coded as “Not specified”.

### Defining eating occasions

Initially, each row of data in the dataset represented a single food item or ingredient consumed by the participant. The total number of food entries was 54,039 over the four day recording period. In order to study the relationship between the eating context at a given meal and FV consumption, food entries that were consumed together as a meal needed to be combined together. A new variable named “eating occasion” (EO) was defined, where food entries with matching answers for variables “subject ID”, “time (hh:mm) of consumption”, “Recording day”, “where”, “with whom”, “TV” and “at table” were aggregated to identify the foods consumed at each EO. The number of EOs generated after aggregation was 16,830.

### Potential confounders

A number of covariates have been identified that may confound the relationship between the eating context and consumption. Consumption behaviour was likely to be different at the seven time points of the day (time slot) and between weekday and weekend days. A binary variable “weekday” was created to differentiate weekdays and weekend days, accounting for potential differences in intake due to different circumstances at weekdays when children were at school or care, versus other activities at the weekend. Children’s age (years) and sex, as well as their socioeconomic status (SES) were also potential confounders. National Statistics Socio-economic Classification (NSSEC) was used as the indicator of SES in this study. NSSEC is the primary social classification used in the UK in all official statistics and surveys [30]. Therefore five covariates: children’s age, sex, NSSEC, timeslot and weekend/weekday variation were adjusted for in the models.

### Statistical analysis

Distributions of categorical variables were described in absolute frequencies and percentages. Nonparametric test for trend was used to test for associations between each category of the eating context variables and the age group (1.5-3y, 4-6y and 7-10y) of children. Fruit consumption and vegetable consumption were analysed separately in all models. Distribution of the amount consumed in both variables across EOs illustrated skewness, due to a high number of EOs where no fruit and/or vegetables were consumed. A two part model was used to analyse the data. Firstly, outcome variables were dichotomised to differentiate EOs where fruit or vegetables were consumed (consumed=1; not consumed=0). Mixed-effects logistic regression was used to estimate the odds ratios (OR) of fruit consumption and vegetable consumption between the subcategories of “where” in relation to the reference subcategory. The same analyses were repeated for “with whom”, “TV” and “at table” respectively. The reference subcategory for each eating context variable was: “At home” for “where”; “Parents only” for “with whom”; “Yes” for “TV on”; and “Yes” for “Eating at table”.

The second part of the model assessed quantities of fruit and vegetables consumed and the four eating context variables in EOs where fruit and/vegetables were consumed, i.e. excluding all EOs with 0g of consumption. EOs were divided into quartiles according to consumption level, with the lowest quartile being the reference category. For fruit, Q1: >0-10g, Q2: >10-50g, Q3: >50-100g, Q4: >100g; and for vegetables, Q1: >0-30g, Q2: >30-60g, Q3: >60-100g, Q4: >100g. Multinomial mixed logistic regression was used for the analyses. The ORs calculated were compared to two reference categories. Firstly, ORs in Q2-4 were compared to the quartile reference (i.e. Q1); secondly, within each quartile, the ORs were compared to the eating context reference category (i.e. “at home” for “where”, “parents only” for “with whom”, “Yes” for “TV on”; and “Yes” for “Eating at table”).

Mixed-effects modelling was used because each participant had repeated measures of EOs over the recording period. This method handles data where observations are not independent of each other and are clustered within group. It is therefore important to correct for this within-person variability (random effect), and the subject ID variable served as the grouping variable in the models. Complete case analyses were performed on all ages and stratified by age groups 1.5-3y, 4-6y and 7-10y, to take into account factors such as schooling and care as well as different stages of growth. “Not specified” responses were treated as missing. All models were adjusted for age, sex, meal time slot and weekday/weekend variations. Analyses were performed using Stata Statistical Software: Release 11 (College Station, TX: StataCorp LP). Only adjusted models are shown in this paper.

## Results

### Characteristics of children and EOs

Table 1 and 2 show the characteristics of participants and EOs. On average, children had four EOs per day. 36.2% of EOs took place at the weekend. There was a similar distribution of EOs in the time slots between 6am and 5pm. The largest proportion of EOs occurred between 5pm-8pm (23.1%), and the fewest between 8pm-6am (8.8%). Of the 16830 EOs, 22.2% were vegetables consuming occasions and 27.6% were fruit. Among these, the median consumption for fruit was 50.0g and 59.6g for vegetables. “Not specified” responses account for 2.1% in the “where” variable, 15.3% in “with whom”, 21.5% in “watching TV” and 23.9% in “sitting at a table”.

**Table 1:** Characteristics of children aged 1.5-10y in NDNS (2008–10)

	**n**	**Mean (S.D)**
**Age**	642	5.3 (2.8)
**Sex**	**n**	**%**
Male	327	50.9
Female	315	49.1
**Age group**		
1.5-3y	219	34.1
4-6y	192	29.9
7-10y	231	36.0
**Mean EOs per day**	**n**	**Mean (S.D)**
1.5-3y	219	4.4 (2.7)
4-6y	192	4.0 (2.3)
7-10y	231	4.0 (2.4)
All ages	642	4.1 (2.5)

**Table 2:** Characteristics of the eating occasions

	**n**	**%**
**Day of Week**		
Monday	2,379	14.1
Tuesday	1,822	10.8
Wednesday	1,511	9.0
Thursday	2,202	13.1
Friday	2,815	16.7
Saturday	3,116	18.5
Sunday	2,985	17.7
**Weekday**	10,729	63.8
**Weekend**	6,101	36.3
**Meal Time Slot**		
6am to 8:59am	2,629	15.6
9am to 11:59am	2,943	17.5
12pm to 1:59pm	2,889	17.2
2pm to 4:59pm	3,000	17.8
5pm to 7:59pm	3,891	23.1
8pm to 5:59pm	1,478	8.8
**EOs consuming**		
Fruit	4,643	27.5
Vegetables	3,734	22.2
FV	7,270	43.2

### Distribution of EOs by where, with whom, TV and at table

Over the diary recording period, children aged 1.5-3y had 80% of EOs at home and 6.6% at care outside home (Table 3). School children aged 4-10y had 69% of EOs at home and 14% at school. Pre-schoolers ate mostly with parents only (48%), whereas older children ate mostly with parents and siblings (≈30%). As age increased, Children ate more frequently with friends (4% 1.5-3y; 16% 4-6y; 19% 7-10y), and alone (4.2% 1.5-3y; 7% 4-6y; 12% 7-10y). The television was off in 61% of the EOs, and children ate at the table (55%) for the majority of the EOs. Trend tests showed significant associations between the age group of children and the four eating context variables (all p<0.05).

**Table 3:** ` by “Where”, “With whom”, “TV on”, “At table”, by age groups, excluding “Not specified” responses

	**All ages**		**1.5-3y**		**4-6y**		**7-10y**		p-trend*
	**n**	**%**	**n**	**%**	**n**	**%**	**n**	**%**	
**Where**									
At home	12,020	**72.9**	4,835	**80.3**	3,315	**68.6**	3,870	**68.8**	<0.001
At school	1,466	**8.9**	**-----**	**-----**	669	**13.9**	775	**13.8**	<0.001
Friend's and relative's house	893	**5.4**	299	**5.0**	251	**5.2**	343	**6.1**	0.007
Care outside home (& school)	495	**3.0**	400	**6.6**	83	**1.7**	**-----**	**-----**	<0.001
Other eateries	340	**2.1**	111	**1.8**	106	**2.2**	129	**2.3**	0.036
Other places	1,265	**7.7**	376	**6.2**	407	**8.4**	510	**9.1**	<0.001
**With whom**									
Parents	4,422	**31.0**	2,528	**47.5**	958	**23.3**	936	**19.4**	<0.001
Parents and siblings	3,816	**26.8**	1,051	**19.8**	1,308	**31.8**	1,457	**30.2**	<0.001
Adult relatives and friends	2,849	**20.0**	1,118	**21.0**	807	**19.6**	924	**19.2**	0.018
Alone	1,077	**7.6**	222	**4.2**	294	**7.1**	561	**11.6**	<0.001
Carer and other children/others	358	**2.5**	216	**4.1**	95	**2.3**	47	**1.0**	<0.001
Friends	1,742	**12.2**	186	**3.5**	656	**15.9**	900	**18.7**	<0.001
**TV on**									
Yes	5,217	**39.5**	2,139	**43.5**	1,412	**37.0**	1,666	**37.1**	--
No	8,002	**60.5**	2,776	**56.5**	2,405	**63.0**	2,821	**62.9**	<0.001
**At table**									
Yes	7,056	**55.1**	2,495	**52.5**	2,163	**58.6**	2,398	**55.0**	--
No	5,748	**44.9**	2,254	**47.5**	1,530	**41.4**	1,964	**45.0**	<0.001

* Nonparametric test for trend across ordered groups was used to test if a trend existed in the distribution of each category and age group of children.

### ”Where” and fruit and vegetable consumption

Compared to eating at home, children of all ages were more likely to consume fruit in EOs at school and at care outside of home, less likely in other eateries and other places (Table 4). They were more likely to consume vegetables at care outside home and at other eateries, but not at other places. When stratified by age group, children aged 1-3y had greater odds of consuming fruit and vegetables at care outside home than at home; school children (4-10y) were most likely to consume fruit at school, to consume vegetables at other eateries than at home.

**Table 4:** Odds of fruit and vegetable consumption by "where" the eating occasions took place, stratified by age group

	**All ages (n=16479)**						**1.5-3y (n=6021)**						**4-6y (n= 4831)**						**7-10y (n=5627)**					
	**Fruit**			**Vegetables**			**Vegetables**			**Vegetables**			**Fruit**			**Vegetables**			**Fruit**			**Vegetables**		
	**OR**	**95% CI**		**OR**	**95% CI**		**OR**	**95% CI**		**OR**	**95% CI**		**OR**	**95% CI**		**OR**	**95% CI**		**OR**	**95% CI**		**OR**	**95% CI**	
At home	1.00			1.00			1.00			1.00			1.00			1.00			1.00			1.00		
At school	**2.98**	2.58	3.45	1.10	0.93	1.31	----	----	----	----	----	----	**3.21**	2.53	4.07	1.31	0.99	1.73	**2.95**	2.34	3.71	1.15	0.88	1.49
Friend's and relative's house	0.97	0.81	1.15	1.10	0.91	1.32	1.03	0.77	1.38	1.19	0.87	1.62	0.88	0.63	1.23	1.26	0.90	1.77	0.96	0.71	1.30	0.92	0.67	1.25
Care outside home	**2.07**	1.67	2.56	**2.15**	1.67	2.78	**2.05**	1.61	2.62	**2.12**	1.59	2.84	**2.02**	1.21	3.38	1.65	0.88	3.08	----	----	----	----	----	----
Other eateries	**0.62**	0.47	0.82	**1.99**	1.54	2.56	0.78	0.50	1.21	1.44	0.91	2.27	0.68	0.42	1.11	**2.36**	1.50	3.72	**0.50**	0.29	0.86	**2.15**	1.42	3.26
Other places	**0.80**	0.69	0.93	**0.27**	0.21	0.34	1.16	0.91	1.48	**0.34**	0.23	0.50	**0.75**	0.58	0.98	**0.22**	0.15	0.34	**0.60**	0.45	0.79	**0.28**	0.20	0.39

All models adjusted for age, sex, timeslot, weekday/weekend variation and NSSEC5.

OR in bold = p < 0.05.

(All eating occasions).

In terms of the quantity consumed, compared to eating at home, children 1.5-3y were more likely to consume between 10g to 100g (Q2 to Q3) of fruit in EOs at care outside home (Q2 OR: 2.39; 95% CI: 1.40-4.07; Q3 OR: 2.12; 95% CI: 1.23-3.63) (Table 5 and Additional file 3: Figure S1). Those aged 4-6y had higher odds of consuming > 50g of fruit at school (Q3 OR: 3.53; 95% CI: 1.95-6.37; Q4 OR: 1.88; 95% CI: 1.06-3.32), and >30-60g of vegetables at school (OR: 3.56; 95% CI: 1.81-7.01) than at home (Additional file 4: Figure S2). Fewer differences were seen in children aged 7-10y, they were less likely to consume >30g of vegetables at other places than at home.

**Table 5:** Odds of fruit and vegetable consumption by quartiles of amount and "where" the eating occasions took place, stratified by age group*

	**All ages**						**1.5-3y**						**4-6y**						**7-10y**					
	**Fruit**			**Vegetables**			**Fruit**			**Vegetables**			**Fruit**			**Vegetables**			**Fruit**			**Vegetables**		
	**n=4542**			**n=3665**			**n=1841**			**n=1259**			**n=1407**			**n=1113**			**n=1294**			**n=1293**		
	**OR**	**95% C.I.**		**OR**	**95% C.I.**		**OR**	**95% C.I.**		**OR**	**95% C.I.**		**OR**	**95% C.I.**		**OR**	**95% C.I.**		**OR**	**95% C.I.**		**OR**	**95% C.I.**	
**Q2**																								
At home	1.00			1.00			1.00			1.00			1.00			1.00			1.00			1.00		
At school	1.41	0.99	2.00	**2.20**	1.48	3.28	----	----	----	----	----	----	1.29	0.71	2.33	**3.56**	1.81	7.01	1.46	0.79	2.71	1.90	0.99	3.64
Friend's and relative's house	0.77	0.50	1.17	1.08	0.72	1.62	1.01	0.52	1.96	1.01	0.53	1.94	0.49	0.22	1.11	1.48	0.69	3.18	0.90	0.41	1.96	0.82	0.39	1.71
Care outside home	**2.35**	1.45	3.81	1.39	0.83	2.34	**2.39**	1.40	4.07	1.60	0.89	2.88	0.98	0.27	3.51	0.29	0.03	2.76	----	----	----	----	----	----
Other eateries	**0.38**	0.19	0.75	**0.58**	0.36	0.93	0.38	0.13	1.07	0.79	0.33	1.90	0.33	0.10	1.10	**0.43**	0.18	1.00	0.65	0.17	2.44	0.62	0.28	1.39
Other places	0.80	0.54	1.19	**0.36**	0.20	0.64	0.72	0.41	1.28	**0.19**	0.05	0.70	0.79	0.40	1.54	**0.30**	0.10	0.94	0.74	0.29	1.86	**0.41**	0.18	0.95
**Q3**																								
At home	1.00			1.00			1.00			1.00			1.00			1.00			1.00			1.00		
At school	**2.55**	1.82	3.58	**1.53**	1.01	2.30	----	----	----	----	----	----	**3.53**	1.95	6.37	1.38	0.69	2.77	1.73	0.95	3.14	**1.91**	1.00	3.65
Friend's and relative's house	0.66	0.43	1.03	1.00	0.66	1.52	1.06	0.55	2.04	0.74	0.36	1.54	**0.19**	0.07	0.54	1.17	0.53	2.56	0.80	0.36	1.79	0.96	0.48	1.93
Care outside home	**2.15**	1.32	3.50	1.00	0.57	1.74	**2.12**	1.23	3.63	0.81	0.41	1.59	2.33	0.71	7.60	1.68	0.49	5.78	----	----	----	----	----	----
Other eateries	**0.37**	0.18	0.74	**0.39**	0.23	0.66	0.60	0.24	1.53	0.43	0.15	1.26	0.37	0.11	1.26	**0.20**	0.07	0.58	0.43	0.10	1.86	0.56	0.25	1.26
Other places	0.91	0.63	1.34	**0.23**	0.12	0.45	0.93	0.54	1.60	0.36	0.12	1.06	0.71	0.36	1.44	**0.30**	0.10	0.93	1.25	0.56	2.81	**0.14**	0.04	0.44
**Q4**																								
At home	1.00			1.00			1.00			1.00			1.00			1.00			1.00			1.00		
At school	**1.87**	1.35	2.60	**0.49**	0.32	0.75	----	----	----	----	----	----	**1.88**	1.06	3.32	**0.49**	0.24	0.98	1.47	0.85	2.55	0.57	0.30	1.09
Friend's and relative's house	0.71	0.47	1.07	0.63	0.41	0.96	1.01	0.51	2.01	0.52	0.23	1.16	0.47	0.22	1.01	0.88	0.41	1.91	0.72	0.35	1.48	**0.50**	0.25	0.99
Care outside home	1.31	0.78	2.19	**0.28**	0.13	0.60	1.34	0.74	2.42	**0.25**	0.10	0.64	1.37	0.43	4.36	0.33	0.07	1.54	----	----	----	----	----	----
Other eateries	**0.21**	0.09	0.47	**0.25**	0.15	0.43	0.55	0.19	1.56	**0.22**	0.06	0.80	**0.15**	0.03	0.62	**0.20**	0.08	0.49	**0.17**	0.03	0.90	**0.32**	0.15	0.70
Other places	1.01	0.70	1.45	**0.14**	0.07	0.27	1.47	0.86	2.50	**0.19**	0.05	0.69	**0.44**	0.22	0.89	**0.11**	0.03	0.42	1.66	0.81	3.41	**0.11**	0.04	0.29

All models adjusted for age, sex, timeslot, weekday/weekend variation and NSSEC.

OR in bold = p < 0.05.

* In eating occasions where fruit or vegetables were consumed only.

### “With whom” and fruit and vegetable consumption

Compared to eating with parents only, children of all ages were more likely to eat both fruit and vegetables with carer and other children/others; more likely to consume fruit with friends; to consume vegetables with parents and siblings and adult family and friends; but least likely to eat vegetables when alone (Table 6). When stratified by age group, the same patterns were found for children aged 1.5-3y and 4-6y, except that those aged 4-6y were also more likely to consume vegetables with friends than with parents. Fewer significant associations were found for children aged 7-10y. They were more likely to eat fruit with friends, but less likely to eat vegetables alone.

**Table 6:** Odds of fruit and vegetable consumption and eating "with whom", stratified by age group

	**All ages (n=14264)**						**1.5-3y (n=5321)**						**4-6y (n=4118)**						**7-10y (n=4825)**					
	**Fruit**			**Vegetables**			**Fruit**			**Vegetables**			**Fruit**			**Vegetables**			**Fruit**			**Vegetables**		
	**OR**	**95% CI**		**OR**	**95% CI**		**OR**	**95% CI**		**OR**	**95% CI**		**OR**	**95% CI**		**OR**	**95% CI**		**OR**	**95% CI**		**OR**	**95% CI**	
Parents	1.00			1.00			1.00			1.00			1.00			1.00			1.00			1.00		
Parent and siblings	1.06	0.94	1.20	**1.35**	1.18	1.56	1.15	0.94	1.40	**1.41**	1.11	1.77	1.13	0.89	1.43	**1.38**	1.06	1.78	0.91	0.72	1.15	1.25	0.96	1.62
Adult relatives and friends	0.99	0.87	1.13	**1.51**	1.32	1.74	1.02	0.85	1.23	**1.39**	1.13	1.71	1.00	0.77	1.29	**1.97**	1.51	2.58	0.91	0.71	1.17	1.22	0.94	1.60
Alone	**0.82**	0.68	1.00	**0.29**	0.22	0.39	0.96	0.67	1.39	**0.27**	0.14	0.50	0.73	0.51	1.06	**0.38**	0.24	0.62	0.78	0.57	1.05	**0.23**	0.15	0.35
Carer and other children/others	**2.70**	2.09	3.48	**2.39**	1.77	3.22	**2.21**	1.59	3.07	**2.80**	1.90	4.11	**3.31**	1.98	5.54	**2.30**	1.28	4.11	**3.34**	1.70	6.56	1.12	0.49	2.57
Friends	**2.14**	1.85	2.48	1.14	0.96	1.35	**1.80**	1.28	2.53	1.38	0.90	2.11	**2.30**	1.77	2.99	**1.70**	1.25	2.30	**2.01**	1.57	2.58	0.87	0.66	1.16

All models adjusted for age, sex, timeslot, weekday/weekend variation and NSSEC.

OR in bold = p < 0.05.

(All eating occasions).

Pre-schoolers (1.5-3y) had higher odds of consuming between >10-100g (Q2 and Q3) of fruit with friends (Q2 OR: 2.69; 95% CI: 1.23-5.88; Q3 OR: 3.49; 95% CI: 1.61-7.57), consuming between >10-50g of fruit with a carer and other children/others (OR: 2.25; 95% CI: 1.15-4.42), and to eat >50-100g of fruit alone (OR: 2.95; 95% CI: 1.06-8.19) than with parents only (Table 7). On the other hand, they were less likely to consume >100g of vegetables with adult relatives and friends than with parents (OR: 0.47; 95% CI: 0.29-0.75). Children 4-6y had a greater odds of consuming >30-60g of vegetables with carer and other children/others (OR: 4.61; 95% CI: 1.28-16.62) and with adult relatives and friends (OR: 2.11; 95% CI: 1.14-3.89) and >50-100g of fruit and >30-60g of vegetables with friends (OR: 1.96; 95% CI: 1.02-3.75 and OR: 3.56; 95% CI: 1.74-7.26 respectively) than with parents. No significant associations were found for children aged 7-10y, except that they were less likely to eat >100g of vegetables with friends than with parents (OR=0.42; 95% CI: 0.22, 0.82).

**Table 7:** Odds of fruit and vegetable consumption by quartiles of amount and eating "with whom", stratified by age groups*

	**All ages**						**1.5-3y**						**4-6y**						**7-10y**					
	**Fruit**			**Vegetables**			**Fruit**			**Vegetables**			**Fruit**			**Vegetables**			**Fruit**			**Vegetables**		
	**n=4035**			**n=3428**			**n=1678**			**n=1189**			**n=1197**			**n=1034**			**n=1160**			**n=1205**		
	**OR**	**95% C.I.**		**OR**	**95% C.I.**		**OR**	**95% C.I.**		**OR**	**95% C.I.**		**OR**	**95% C.I.**		**OR**	**95% C.I.**		**OR**	**95% C.I.**		**OR**	**95% C.I.**	
**Q2**																								
Parents	1.00			1.00			1.00			1.00			1.00			1.00			1.00			1.00		
Parent and siblings	1.08	0.81	1.43	1.09	0.80	1.47	0.67	0.44	1.02	1.01	0.63	1.61	1.75	1.00	3.07	1.48	0.82	2.69	1.26	0.68	2.33	1.13	0.62	2.05
Adult relatives and friends	0.95	0.70	1.28	1.16	0.86	1.56	0.98	0.65	1.47	0.85	0.56	1.30	1.43	0.76	2.68	**2.11**	1.14	3.89	0.54	0.28	1.07	1.46	0.79	2.71
Alone	1.34	0.80	2.25	0.66	0.30	1.42	2.38	0.85	6.69	0.29	0.03	2.79	1.93	0.68	5.51	0.86	0.22	3.29	0.84	0.36	1.94	0.86	0.28	2.63
Carer and other children/others	**2.57**	1.49	4.43	1.76	0.98	3.16	**2.25**	1.15	4.42	1.49	0.72	3.11	3.42	0.98	11.94	**4.61**	1.28	16.62	1.59	0.37	6.79	0.78	0.11	5.69
Friends	1.28	0.90	1.83	**1.76**	1.20	2.58	**2.69**	1.23	5.88	1.30	0.54	3.13	1.00	0.52	1.95	**3.56**	1.74	7.26	1.17	0.58	2.33	1.47	0.73	2.94
**Q3**																								
Parents	1.00			1.00			1.00			1.00			1.00			1.00			1.00			1.00		
Parent and siblings	1.06	0.80	1.42	0.96	0.71	1.30	0.77	0.51	1.17	0.77	0.47	1.26	1.46	0.82	2.60	1.07	0.60	1.90	1.21	0.64	2.31	1.19	0.66	2.16
Adult relatives and friends	1.02	0.75	1.39	0.99	0.73	1.34	1.11	0.73	1.68	0.66	0.43	1.03	1.09	0.57	2.10	1.37	0.75	2.49	0.85	0.43	1.66	1.47	0.80	2.70
Alone	1.58	0.95	2.63	0.60	0.28	1.27	**2.95**	1.06	8.19	0.78	0.16	3.81	2.06	0.73	5.80	0.45	0.11	1.95	1.05	0.46	2.43	0.90	0.29	2.78
Carer and other children/others	**2.11**	1.20	3.69	0.96	0.50	1.84	1.89	0.94	3.78	0.53	0.22	1.30	2.40	0.65	8.82	2.43	0.67	8.89	2.60	0.62	10.85	1.01	0.16	6.32
Friends	**2.18**	1.54	3.09	1.11	0.74	1.65	**3.49**	1.61	7.57	0.69	0.26	1.84	**1.96**	1.02	3.75	1.28	0.61	2.68	1.99	0.99	3.98	1.38	0.69	2.77
**Q4**																								
Parents	1.00			1.00			1.00			1.00			1.00			1.00			1.00			1.00		
Parent and siblings	0.95	0.71	1.26	0.95	0.71	1.27	0.67	0.43	1.04	0.87	0.54	1.41	1.25	0.72	2.19	0.82	0.48	1.39	1.04	0.59	1.84	1.16	0.69	1.95
Adult relatives and friends	0.91	0.67	1.23	0.85	0.63	1.13	0.96	0.62	1.48	**0.47**	0.29	0.75	1.22	0.66	2.27	1.36	0.79	2.35	0.61	0.33	1.10	1.07	0.62	1.83
Alone	1.17	0.71	1.92	0.54	0.27	1.07	1.80	0.62	5.24	0.83	0.17	4.08	1.86	0.68	5.11	0.59	0.18	1.93	0.78	0.37	1.63	0.59	0.22	1.59
Carer and other children/others	1.20	0.67	2.14	**0.47**	0.22	0.99	0.94	0.44	2.03	0.38	0.13	1.12	1.89	0.54	6.67	0.37	0.08	1.80	0.77	0.18	3.21	0.67	0.12	3.73
Friends	1.34	0.96	1.89	**0.44**	0.29	0.66	1.06	0.43	2.63	0.45	0.14	1.37	1.32	0.70	2.48	0.58	0.29	1.18	1.32	0.71	2.46	**0.42**	0.22	0.82

All models adjusted for age, sex, timeslot, weekday/weekend variation and NSSEC.

OR in bold = p < 0.05.

* In eating occasions where fruit or vegetables were consumed only.

### “TV on” and fruit and vegetable consumption

Across all ages, as well as by stratum, children were more likely to eat vegetables when the TV was off (Table 8). Few associations were found between the quantity consumed and the TV on or off, except for weak associations with consuming >100g of fruit in children 1.5-3y and 4-6y (data not shown).

**Table 8:** Odds of fruit and vegetable consumption and TV on/off, stratified by age group

	**All ages (n=13219)**						**1.5-3y (n=4915)**						**4-6y (n=3817)**						**7-10y (n=4487)**					
	**Fruit**			**Vegetables**			**Fruit**			**Vegetables**			**Fruit**			**Vegetables**			**Fruit**			**Vegetables**		
	**OR**	**95% CI**		**OR**	**95% CI**		**OR**	**95% CI**		**OR**	**95% CI**		**OR**	**95% CI**		**OR**	**95% CI**		**OR**	**95% CI**		**OR**	**95% CI**	
TV on	1.00			1.00			1.00			1.00			1.00			1.00			1.00			1.00		
TV off	0.98	0.89	1.08	**1.51**	1.35	1.68	0.94	0.81	1.09	**1.63**	1.36	1.95	1.03	0.87	1.23	**1.53**	1.24	1.88	1.00	0.84	1.19	**1.47**	1.22	1.77

All models adjusted for age, sex, timeslot, weekday/weekend variation and NSSEC.

OR in bold = p < 0.05.

(All eating occasions).

### “Eating at a table” and fruit and vegetable consumption

In terms of eating at a table, children aged 1.5-3y and 4-6y were less likely to consume fruit or vegetables when they were not eating at a table (Table 9). The associations were stronger for vegetables than for fruit. For children aged 7-10y, no difference was found for fruit; however they were less likely to consume vegetables when not eating at a table.

**Table 9:** Odds of fruit and vegetable consumption and "eating at table", stratified by age group

	**All ages (n=12804)**						**1.5-3y (n=4749)**						**4-6y (n=3493)**						**7-10y (n=4362)**					
	**Fruit**			**Vegetables**			**Fruit**			**Vegetables**			**Fruit**			**Vegetables**			**Fruit**			**Vegetables**		
	**OR**	**95% CI**		**OR**	**95% CI**		**OR**	**95% CI**		**OR**	**95% CI**		**OR**	**95% CI**		**OR**	**95% CI**		**OR**	**95% CI**		**OR**	**95% CI**	
At table	1.00			1.00			1.00			1.00			1.00			1.00			1.00			1.00		
Not at table	**0.69**	0.63	0.76	**0.11**	0.10	0.13	**0.58**	0.50	0.66	**0.10**	0.08	0.12	**0.70**	0.59	0.83	**0.16**	0.13	0.20	0.92	0.78	1.09	**0.09**	0.07	0.12

All models adjusted for age, sex, timeslot, weekday/weekend variation and NSSEC.

OR in bold = p < 0.05.

(All eating occasions).

When quantifying fruit and vegetable consumption at the table, opposite trends were seen between fruit and vegetables. Children were more likely to consume large amounts (>100g) of fruit when not eating at a table (Table 10). Those aged 7-10y had significantly higher odds of consuming >10g (Q2, Q3 and Q4) of fruit when not eating at a table (Q2 OR: 1.75; 95% CI: 1.06-2.87; Q3 OR: 1.73; 95% CI: 1.07-2.82; Q4 OR: 2.01; 95% CI: 1.29-3.13). On the contrary, not eating at a table greatly reduced the odds of consuming all amounts of vegetables in children, especially in the 7-10y age group.

**Table 10:** Odds of fruit and vegetable consumption by quartiles of amount and "eating at table", stratified by age group*

	**All ages**						**1.5-3y**						**4-6y**						**7-10y**					
	**Fruit**			**Vegetables**			**Fruit**			**Vegetables**			**Fruit**			**Vegetables**			**Fruit**			**Vegetables**		
	**n=3636**			**n=3119**			**n=1512**			**n=1065**			**n=1439**			**n=1134**			**n=1022**			**n=1080**		
	**OR**	**95% C.I.**		**OR**	**95% C.I.**		**OR**	**95% C.I.**		**OR**	**95% C.I.**		**OR**	**95% C.I.**		**OR**	**95% C.I.**		**OR**	**95% C.I.**		**OR**	**95% C.I.**	
**Q2**																								
At table	1.00			1.00			1.00			1.00			1.00			1.00			1.00			1.00		
Not at table	1.23	0.97	1.55	**0.45**	0.33	0.62	1.15	0.82	1.60	**0.54**	0.34	0.88	1.02	0.65	1.60	**0.49**	0.27	0.88	**1.75**	1.06	2.87	**0.36**	0.20	0.65
**Q3**																								
At table	1.00			1.00			1.00			1.00			1.00			1.00			1.00			1.00		
Not at table	**1.42**	1.13	1.79	**0.65**	0.48	0.87	1.37	0.98	1.91	0.63	0.39	1.04	1.29	0.83	2.01	1.02	0.61	1.73	**1.73**	1.07	2.82	**0.40**	0.22	0.71
**Q4**																								
At table	1.00			1.00			1.00			1.00			1.00			1.00			1.00			1.00		
Not at table	**1.70**	1.36	2.14	**0.44**	0.33	0.60	**1.60**	1.13	2.27	**0.43**	0.25	0.75	**1.73**	1.13	2.64	0.69	0.41	1.17	**2.01**	1.29	3.13	**0.27**	0.16	0.46

All models adjusted for age, sex, timeslot, weekday/weekend variation and NSSEC.

OR in bold = p < 0.05.

* In eating occasions where fruit or vegetables were consumed only.

## Discussion

In this study we demonstrated a new methodology for measuring the eating context of young children and related it to fruit and vegetable consumption in a nationally representative sample of UK children. The results provide new insights into how fruit and/or vegetable consumption varies in different eating locations, with different people present, whether or not the TV is on and if eaten at a table. The results from this sample of children suggested that they were more likely to consume higher amounts of fruit and vegetables in structured and formal care settings, such as school and care outside of home than at home, and with carers and other children and their friends, than compared to the EOs taking place with parents alone. These associations were particularly strong for the younger age groups (1.5-3y and 4-6y). Fewer significant associations were seen for older children.

There are a number of possible explanations for the higher fruit and vegetable consumption seen in structured care and school settings. In 2005, the Department of Education and Skills produced guidelines to implement nutrition standards for lunches in nursery schools, which included a recommendation that an item of fruit and vegetables to be provided to each child at lunchtime each day [31]. The Caroline Walker Trust published nutrient-based standards for children under 5 years in child care, which recommended that carers offer 4 to 5 different types of fruit and vegetables to children each day in day care settings [32]. For school children, the School Meals Review Panel was appointed by the UK government in 2005 to introduce new standards for school food, whereby schools had to provide at least one portion of fruit and one portion of vegetables or salad at lunch per pupil per day [33]. Furthermore, children aged 4-6y in England who attend local education authority (LEA) maintained schools are entitled to a free piece of fruit or vegetables each school day [34], which may explain the higher odds ratios for fruit (Q3) and vegetables (Q2) in this age group, which were not seen in the 7-10y age group. These changes in recommendations may well explain the increased consumption in school and formal care settings.

Older children (7-10y) had a greater tendency to eat with friends as well as alone compared to younger children, thus illustrating the gain in independence and the increasing importance of peers in eating and food choice as they become older [35,36]. A number of intervention studies have shown that children have increased consumption of, or have expressed a liking of fruit and vegetables, due to the influence of their peers eating or liking fruit and vegetables [37-39]. Our observational study showed that children were consuming more fruit and vegetables when they were with friends than with parents, suggesting a possible effect of peer modelling on consumption of healthy foods. However, higher consumption with friends could also be confounded by the location of the eating occasions such as school or care settings. This is a speculation since we did not test the combined effects of the eating contexts on consumption. Having the TV on appeared to influence the odds of consuming vegetables, but was not associated with the quantity consumed. No strong associations were seen for fruit. Our findings only partially agree with previous studies, where fruit and vegetable consumption has been found to be inversely correlated with the frequency or number of hours of TV watching [22,40,41]. Our results are based on data for each eating occasion and may therefore capture differences in behaviour between fruit consumption and vegetable consumption, which may not be seen using questionnaires as in the other studies of the influence of television.

This study also exhibited new information on eating at the table and fruit and vegetable consumption, since this determinant has not been well researched. Children who ate at the table were much more likely to eat vegetables. This was not surprising, since previous studies have shown that family meal frequency is important in establishing positive eating behaviours such as eating fruit and vegetables, and family meals are more likely to occur around a table [42-45]. However, interesting findings were seen when quantifying the amount consumed when eating at the table. While the first part of the model suggested that children were more likely to consume fruit at the table, the second part of the model indicated large portions of fruit (>100g) were consumed when not at a table. We are unsure of the reason for this observation, but a possible explanation could be that the higher odds of fruit consumption in the first part of the model were driven by higher frequency of occasions consuming small amounts of fruit in composite dishes (such as fruit puddings and desserts) at the table; whilst results in the second part of the model might have been driven by fruit in large discrete portions, such as whole apples and bananas, with typical portion sizes of around 100g, that were likely to be eaten on the go and in any environment without being at the table. Unlike fruit, vegetables were less likely to be consumed in large quantities when not at the table. This is because vegetables are usually served as part of a main meal in substantial portions rather than eaten as snacks, and hence vegetables are more likely to be eaten at a table. These findings emphasise that eating fruit and eating vegetables are different behaviours.

There are a number of strengths of this study. It illustrates an alternative methodology for assessing the eating environment using a simple traditional dietary assessment tool – a food diary. The advantage of this method is that it is easy to use, and collects real time information without using advanced technology, which remains important for national surveys where there are different levels of technology access and expertise and the wide ranges of age and sociodemographic background among participants. The burden of collecting the information is also low, since it can be combined as part of an existing dietary assessment tool (food diary or 24 hour recall) without using a separate questionnaire or other psychometric measure. Previously, data on the food environment, such as television watching and screen time and family meal frequency have been collected via questionnaires, while dietary intake was assessed using separate dietary assessment method. Our method is likely to be more accurate since we have detailed dietary data that match with the eating contexts for each eating occasion. Other studies had investigated specific meals such as school lunches using direct observation methods; the method described here dispenses with interviewer burden as well as the discomfort for participants of being watched and monitored.

This study has its limitations however. Children’s fruit and vegetable consumption and the eating context information were reported by their parents. Previous studies have shown low agreements between children and parental reports of fruit and vegetable intakes [46-48], and also parents had to rely on information given by other carers if the children had been away from home. Hence, there could be errors induced during the recording process. Secondly, there were a number of “Not specified” responses when collecting information on the four eating context elements, particularly in answering whether the TV was on, and eating at the table. This may be because the children were in eating contexts where televisions or tables were not present, for instance, in outdoor areas or when eating on the go. These “Not specified” responses were treated as missing data because it would be bias to make assumptions on these data. The fact that only complete case analyses were performed may have resulted in reduced statistical power to detect differences amongst categories, as the number of eating occasions included in the analyses was lowered. Another limitation was that parents were only asked to record if TV was on at the occasion, it was unknown whether the children were actively watching TV during the meal and how much distraction from the TV might have influenced consumption. Moreover, NDNS did not collect data on other sociodemographic measures such as parental education that might have affected consumption. These factors were not adjusted for in the analysis and hence, there could be residual confounding in the results.

Furthermore, this study could not determine the direction of the effects between the eating context and fruit and vegetable consumption and if the effects were causal, because of the cross sectional design of the NDNS survey. For instance, it is possible that fruit and vegetable consumption is driven by the eating contexts, such as the location of the meal, but there may also be circumstances where eating contexts are driven by food choices or consumption. Further research should therefore be done longitudinally to allow clearer causality conclusions to be drawn. In addition, this new method of eating context assessment needs validation of its accuracy as we have only focused on one food group as dietary outcome, it should also be re-tested on other populations and sub-populations, as well as on other dietary assessment tools such as 24 hour recall. Relationships between the eating context and other dietary outcomes such as energy dense food and drinks should be studied as they may also be related to the eating environment. We intend to apply this assessment method to Year 3 and 4 of the rolling programme for validity as well as testing it on other age groups and food groups. Lastly, the four eating context elements in this study have been analysed separately and this limits the understanding the relative weight of each element and the combined effect of the eating contexts on overall consumption. Hence, for future work, these factors could be modelled in such a way that their association with the outcome as well as with one another can be determined.

## Conclusions

This study illustrated a new way of capturing and assessing various eating contexts using current dietary assessment tools at the population level. In this sample population, we found that children were more likely to consume fruit and vegetables at structured and regulated settings, such as schools and formal child care than at home, as well as eating at a table and with TV off. The results may encourage parents and practitioners to create and promote positive social eating environments and to provide better food choices for children, particularly at home. Researchers should also consider the eating contexts when designing future programmes and interventions that target dietary behaviours and food consumption.

## Abbreviations

CI: Confidence interval; DINO: Diet in nutrients out; EOs: Eating occasions; EMA: Ecological momentary Aassessment; FSA: Food standards agency; FV: Fruit and vegetables; LEA: Local education authority; NDB: Nutrient databank; NDNS: National diet and nutrition survey; NSSEC: National statistics socio-economic classification; OR: Odds ratio; SES: Socio-economic status.

## Competing interests

The authors declare no competing interests.

## Authors’ contributions

TNM developed the methodology, analysed and interpreted the data, and wrote the manuscript. CJP and TNM prepared the dietary data for analysis. TNM prepared the eating context data. AMS initiated the study idea, oversaw the NDNS dietary data collection, coding and analysis, supervised and edited the manuscript. All authors read and approved the final manuscript.

## Supplementary Material

Additional file 1 **Appendix 1.** An extract of food diary page used in NDNS to illustrate assessment of eating context and dietary intake. Click here for file

Additional file 2 **Appendix 2.** List of subgroups within the Where and Who categories. Click here for file

Additional file 3 **Figure S1.** Illustration of odds ratios of consuming fruit by quartiles at different eating locations in all ages of children and stratified by age groups 1.5-3y, 4-6y and 7-10y. Quartile 1 (>0-10g) is the reference quartile category, and "At home" is the reference category for the "where" eating context variable. *p<0.05, **p<0.01, ***p<0.001. Click here for file

Additional file 4 **Figure S2. **Illustration of odds ratios of consuming vegetables by quartiles at different eating locations in all ages of children and stratified by age groups 1.5-3y, 4-6y and 7-10y. Quartile 1 (>0-30g) is the reference quartile category, and "At home" is the reference category for the "where" eating context variable. *p<0.05, **p<0.01, ***p<0.001. Click here for file
