# Supplementary material for: Assessing eating context and fruit and vegetable consumption in children: new methods using food diaries in the UK National Diet and Nutrition Survey Rolling Programme
Source: Int J Behav Nutr Phys Act. 2012 Oct 18;9:126. doi: 10.1186/1479-5868-9-126 (PMC3495842; doi:10.1186/1479-5868-9-126)

**Appendix 1. An extract of food diary page used in NDNS to illustrate assessment of eating context and dietary intake.**
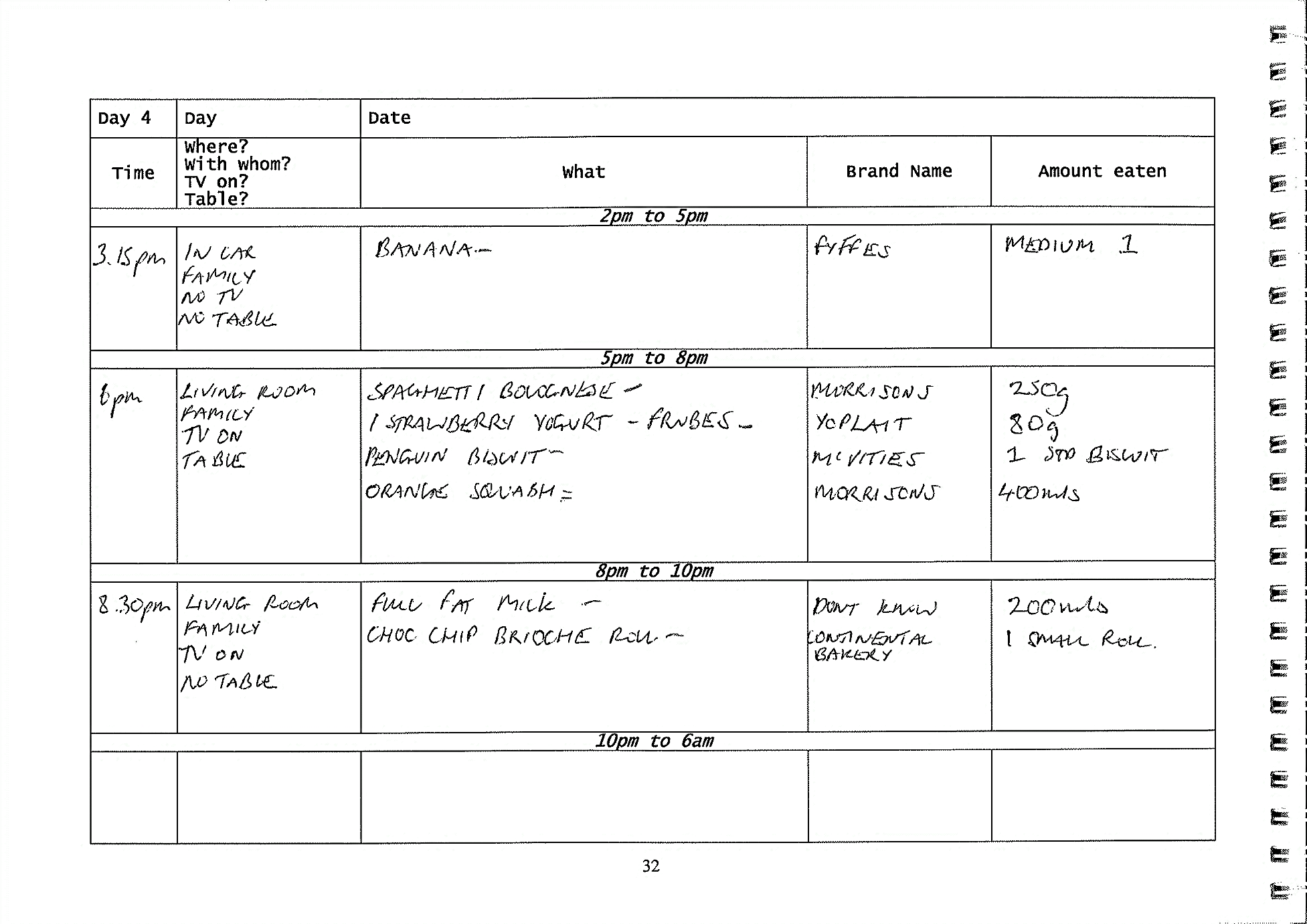

Supplement: Additional file 1 — Appendix 1. An extract of food diary page used in NDNS to illustrate assessment of eating context and dietary intake. [file 1479-5868-9-126-S1.docx]
