# Supplementary material for: Assessing eating context and fruit and vegetable consumption in children: new methods using food diaries in the UK National Diet and Nutrition Survey Rolling Programme
Source: Int J Behav Nutr Phys Act. 2012 Oct 18;9:126. doi: 10.1186/1479-5868-9-126 (PMC3495842; doi:10.1186/1479-5868-9-126)
Supplement: Additional file 2 — Appendix 2. List of subgroups within the Where and Who categories. [file 1479-5868-9-126-S2.docx]

| Appendix 2. List of subgroups within the Where and Who categories. | |
| --- | --- |
| Where |  |
| At home | Home – Bedroom |
|  | Home - Dining Room |
|  | Home - Garden |
|  | Home - Kitchen |
|  | Home - Living Room |
|  | Home - Other |
| At school | School - Canteen - Bought food |
| (Age 4-10y only) | School - Canteen - Food from home |
|  | School - Canteen - Other |
|  | School - Classroom |
|  | School - Other |
|  | School - Playground |
| Friend's or relative's house | Friend's or Relative's house |
| Care outside home | Community Centre/Day Centre/Drop in |
| (Age 1.5-3y; 4-6y only) | Carer's home |
|  | Nursery/Kindergarten |
| Other eateries | Coffee shop, café, shop, deli, sandwich bar |
|  | Fast food outlet |
|  | Restaurant, pub, night club |
|  | Work - Canteen - Bought food |
|  | Work - Canteen - Food from home |
| Other places | Bus, car, train |
|  | Holiday Accommodation |
|  | Leisure Activities, shopping, tourist attraction, cinema |
|  | Other place |
|  | Outside - Other |
|  | Place of Worship |
|  | Public Hall/Function Room |
|  | Sports club, sports leisure venue |
|  | Street |
|  | Work - Other |
| Not specified | Participant did not specify |
| With whom |  |
| \| **Parents** \| \| --- \| \| **Parents & siblings** \| \|  \| \| **Adult relatives & friends** \| \|  \| \| **Alone** \| \| **Carer and other children/ others** \| \|  \| \|  \| \| **Friends** \| \| **Not specified** \| | \| Parent(s)/Long term carer(s) \| \| --- \| \| Parent(s)/ Long term carer(s)& Siblings \| \| Siblings \| \| Family (incl. Relatives) \| \| Family & Friends \| \| Alone \| \| Child/Children \| \| Carer & Other Children \| \| Others \| \| Friends \| \| Participant did not specify \| \|  \| |
