# Supplementary figures and images for: Assessing eating context and fruit and vegetable consumption in children: new methods using food diaries in the UK National Diet and Nutrition Survey Rolling Programme
Source: Int J Behav Nutr Phys Act. 2012 Oct 18;9:126. doi: 10.1186/1479-5868-9-126 (PMC3495842; doi:10.1186/1479-5868-9-126)

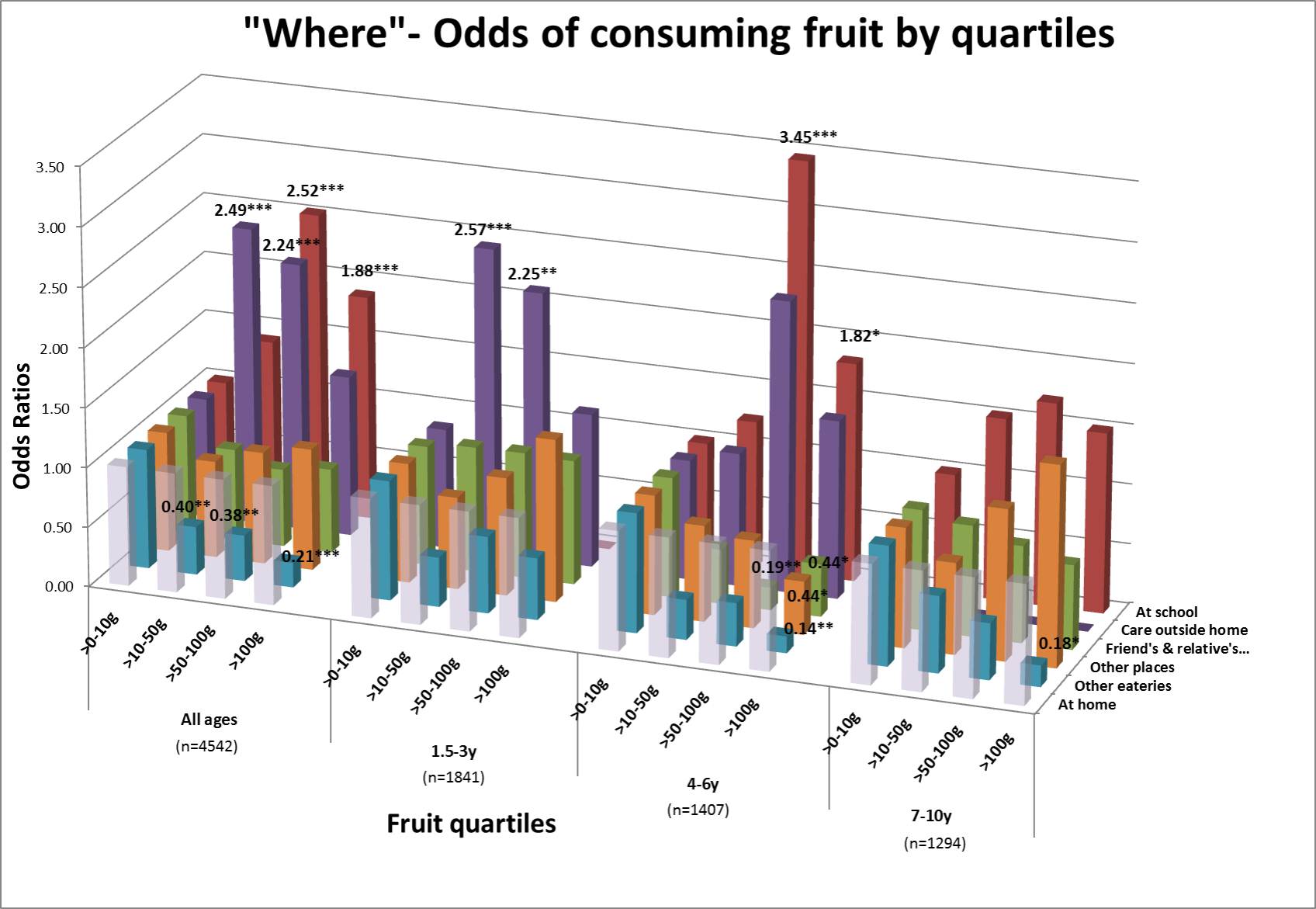

Supplement: Additional file 3 — Figure S1. Illustration of odds ratios of consuming fruit by quartiles at different eating locations in all ages of children and stratified by age groups 1.5-3y, 4-6y and 7-10y. Quartile 1 (>0-10g) is the reference quartile category, and "At home" is the reference category for the "where" eating context variable. *p<0.05, **p<0.01, ***p<0.001. [file 1479-5868-9-126-S3.jpeg]

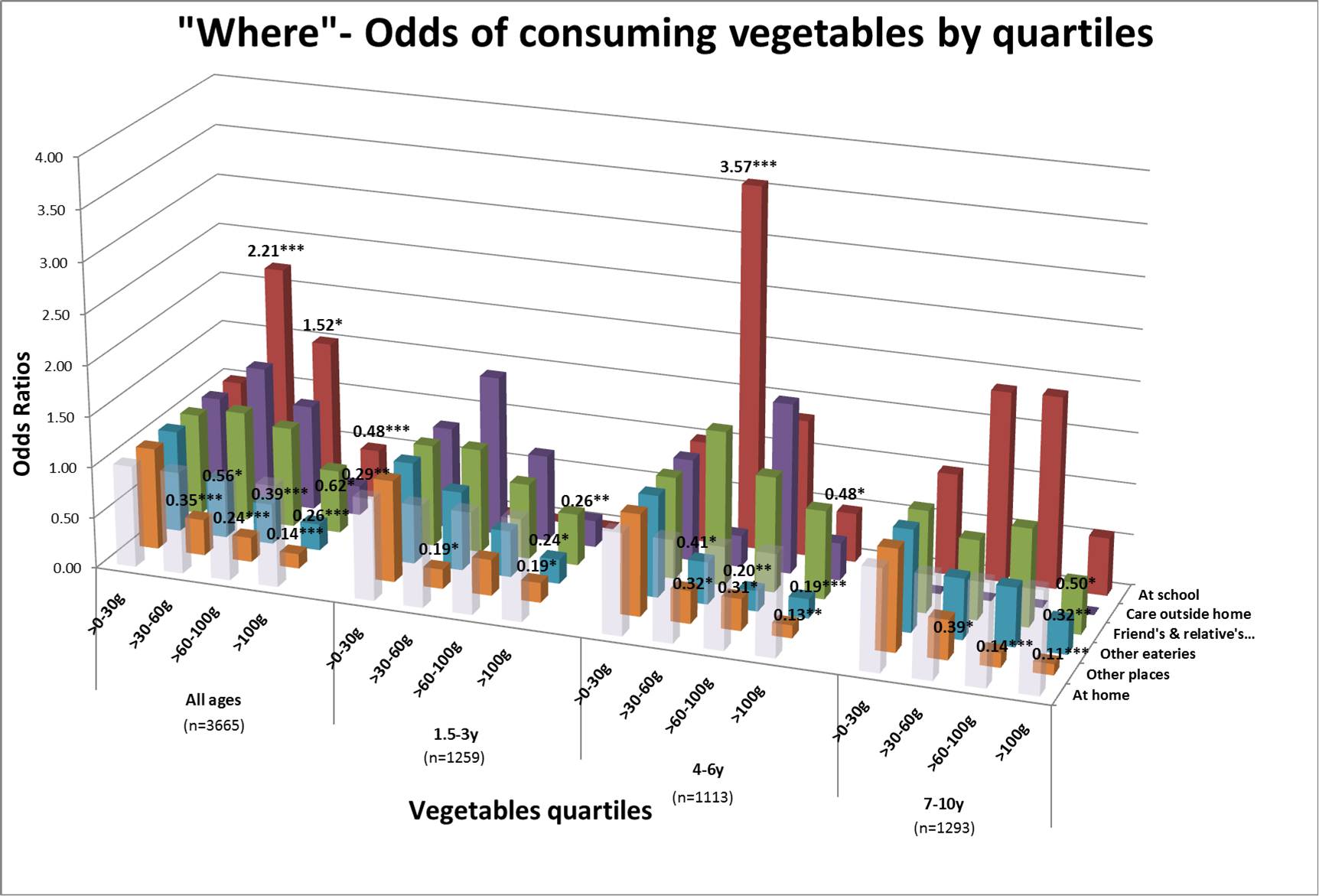

Supplement: Additional file 4 — Figure S2. Illustration of odds ratios of consuming vegetables by quartiles at different eating locations in all ages of children and stratified by age groups 1.5-3y, 4-6y and 7-10y. Quartile 1 (>0-30g) is the reference quartile category, and "At home" is the reference category for the "where" eating context variable. *p<0.05, **p<0.01, ***p<0.001. [file 1479-5868-9-126-S4.jpeg]
